# Supplementary material for: Learning properties of quantum states without the IID assumption
Source: Nat Commun. 2024 Nov 8;15:9677. doi: 10.1038/s41467-024-53765-6 (PMC11549401; doi:10.1038/s41467-024-53765-6)
Supplement: Supplementary file 1 — Supplementary Information [file 41467_2024_53765_MOESM1_ESM.pdf]

Supplementary Information for: Learning Properties of Quantum States Without the IID Assumption

Omar Fawzi[1], Richard Kueng[2], Damian Markham[3], and Aadil Oufkir[1,4]

[1] *Univ Lyon, Inria, ENS Lyon, UCBL, LIP, Lyon, France*
[2] *Institute for Integrated Circuits, Johannes Kepler University Linz, Altenberger Straße 69, Austria*
[3] *Sorbonne Université, CNRS, LIP6, F-75005 Paris, France*
[4] *Institute for Quantum Information, RWTH Aachen University, Aachen, Germany*

Supplementary Note 1 – Conditioning on the permutation renders basic learning tasks impossible

Consider the following classical problem $\mathrm{SUCCESS}_\varepsilon = \{(p,\sigma) \in [0,1]\times \mathrm{D}(\{0,1\}) : |p-\sigma(1)| \le \varepsilon\}$. Here, $\mathrm{D}(\{0,1\})$ refers to the set of probability distributions over $\{0,1\}$. A strategy in this case is described by a possibly random function $F : \{0,1\}^{N-1} \to [0,1]$. Recall the definition of error probability in Equation (2) of the main text

$$\delta'_{\mathcal{B}}(N, \rho^{A_1\cdots A_N}, \varepsilon) = \mathbb{E}_\pi \left[\mathbb{P}_{(c,p)\sim\mathcal{B}(\rho^\pi)}\left[\left(p, (\rho^\pi)^{A_N}_{c,p}\right) \notin \mathrm{SUCCESS}_\varepsilon\right]\right].$$

Assume we have for any distribution $P^{A_1\cdots A_N}$

$$\mathbb{E}_\pi \left[\delta'_F(N, P^\pi, \varepsilon)\right] \le \delta. \tag{1}$$

Consider the distribution putting mass on a single string $x_1, \dots, x_N$. Then Supplementary Equation (1) can be written as

$$\mathbb{E}_\pi \left[\mathbb{P}\left[|x_{\pi(N)} - F(x_{\pi(1)}, x_{\pi(2)}, \dots, x_{\pi(N-1)})| > \varepsilon\right]\right] \le \delta. \tag{2}$$

Now, assume N is even and let $x_i = 0$ for $i \le N/2$ and $x_i = 1$ for $i > N/2$. We write

$$\begin{aligned}
\delta &\ge \mathbb{P}\left[|x_{\pi(N)} - F(x_{\pi(1)}, x_{\pi(2)}, \dots, x_{\pi(N-1)})| > \varepsilon\right] \\
&= \mathbb{P}\left[\pi(N) \le N/2\right]\mathbb{P}\left[|0 - F(x_{\pi(1)}, x_{\pi(2)}, \dots, x_{\pi(N-1)})| > \varepsilon | \pi(N) \le N/2\right] \\
&\quad + \mathbb{P}\left[\pi(N) > N/2\right]\mathbb{P}\left[|1 - F(x_{\pi(1)}, x_{\pi(2)}, \dots, x_{\pi(N-1)})| > \varepsilon | \pi(N) > N/2\right] \\
&\ge \frac{1}{2}\mathbb{P}\left[|1 - F(x_{\pi(1)}, x_{\pi(2)}, \dots, x_{\pi(N-1)})| > \varepsilon | \pi(N) > N/2\right].
\end{aligned} \tag{3}$$

Note that conditioned on $\pi(N) > N/2$, the string $(x_{\pi(1)}, x_{\pi(2)}, \dots, x_{\pi(N-1)})$ is uniformly distributed on all bitstrings that have $N/2$ zeros and $N/2-1$ ones. We can also apply the success criterion in Supplementary Equation (2) for the bitstring $x_i = 0$ for $i \le N/2+1$ and $x_i = 1$ for $i > N/2+1$ which gives

$$\delta \ge \frac{N/2+1}{N}\mathbb{P}\left[|F(x_{\pi(1)}, x_{\pi(2)}, \dots, x_{\pi(N-1)})| > \varepsilon | \pi(N) \le N/2+1\right]. \tag{4}$$

Note that we also have that conditioned on $\pi(N) \le N/2+1$, the string $(x_{\pi(1)}, x_{\pi(2)}, \dots, x_{\pi(N-1)})$ is uniformly distributed on all bitstrings that have $N/2$ zeros and $N/2-1$ ones. Let $Y_1, \dots, Y_{N-1}$ be a random variable chosen according to this distribution. Then, Supplementary Equation (3) and Supplementary Equation (4) can be expressed as $\mathbb{P}\left[|1 - F(Y_1, Y_2, \dots, Y_{N-1})| > \varepsilon\right] \le 2\delta$ and $\mathbb{P}\left[|F(Y_1, Y_2, \dots, Y_{N-1})| > \varepsilon\right] \le \frac{N}{N/2+1}\delta \le 2\delta$ respectively. For $\varepsilon < 1/2$, the events $|1 - F(Y_1, Y_2, \dots, Y_{N-1})| \le \varepsilon$ and $|F(Y_1, Y_2, \dots, Y_{N-1})| \le \varepsilon$ are disjoint, this implies that $\delta \ge 1/4$.

Supplementary Note 2 – Illustration of the randomized local de Finetti Theorem 4

In this section, we illustrate Theorem 4 of the main text for a specific permutation invariant state and a specific distribution of measurements. Recall Theorem 4 of the main text:

Theorem (Randomized local de Finetti). Let $N \geq 1$ be a positive integer and $A_1 \cong A_2 \cong \cdots \cong A_N$ be N isomorphic quantum systems of dimension d. Let $1 \leq k < \sqrt{\frac{N}{\log(d)}}$. Let $\rho^{A_1\cdots A_N}$ be a state and let q^N be a permutation-invariant measure on $\mathcal{R}^N$. Let $\{\Lambda_r\}_{r\in\mathcal{R}}$ be a set of measurement channels with input system A and output system X. Let $\mathbf{j} = (j_1, \dots, j_N)$ be a random permutation of $\{1, \dots, N\}$, $l \sim \mathrm{Unif}\{k+1, \dots, k+\frac{N}{2}\}$, $\mathbf{r} = (r_1, \dots, r_N) \sim q^N$ and $\mathbf{w} = (w_{l+1}, \dots, w_{k+N/2})$ be the outcomes of measuring the systems $A_{j_{l+1}}, \dots, A_{j_{k+N/2}}$ using the measurements $\Lambda_{r_{l+1}}, \dots, \Lambda_{r_{k+N/2}}$. The following inequality holds:

$$\mathbb{E}_{\mathbf{j},l,\mathbf{r}\sim q^N}\left[\sum_{\mathbf{w}} p_{\mathbf{r}}(\mathbf{w})\left\|\mathrm{id}\otimes\left(\bigotimes_{i=2}^{k+1}\Lambda_{r_i}\right)\left(\rho_{l,\mathbf{r},\mathbf{w}}^{A_{j_1}\cdots A_{j_{k+1}}} - \bigotimes_{i=1}^{k+1}\rho_{l,\mathbf{r},\mathbf{w}}^{A_{j_i}}\right)\right\|_1\right] \leq \sqrt{\frac{4k^2\log(d)}{N}},$$

where $p_{\mathbf{r}}(\mathbf{w}) = \mathrm{Tr}\left[\langle\mathbf{w}|(\Lambda_{r_{l+1}}\otimes\cdots\otimes\Lambda_{r_{k+N/2}})(\rho^{A_{j_1}\cdots A_{j_N}})|\mathbf{w}\rangle\right]$ and we defined the conditional state $\rho_{l,\mathbf{r},\mathbf{w}}$ as

$$\rho_{l,\mathbf{r},\mathbf{w}}^{A_{j_1}\cdots A_{j_{k+1}}} = \frac{1}{p_{\mathbf{r}}(\mathbf{w})}\mathrm{Tr}_{A_{j_{k+2}}\cdots A_{j_N}}\left[\langle\mathbf{w}|(\Lambda_{r_{l+1}}\otimes\cdots\otimes\Lambda_{r_{k+N/2}})(\rho^{A_{j_1}\cdots A_{j_N}})|\mathbf{w}\rangle\right].$$

Note that if $\rho^{A_1\cdots A_N}$ is permutation invariant, the random permutation $\mathbf{j}$ is not needed and we can replace j_i by i and $\bigotimes_{i=1}^{k+1}\rho_{l,\mathbf{r},\mathbf{w}}^{A_{j_i}}$ by $\left(\rho_{l,\mathbf{r},\mathbf{w}}^{A_N}\right)^{\otimes k+1}$ in the above expressions.

Supplementary Example 1. Let the dimension be $d = 2$. The quantum state we consider is

$$\rho = \int \mathrm{dHaar}(\varphi)|\varphi\rangle\langle\varphi|^{\otimes N}.$$

The law of measurement devices is the Dirac delta distribution on the simple POVM $\mathcal{M} = \{|0\rangle\langle 0|, |1\rangle\langle 1|\}$. Observe that any Dirac delta distribution of measurement devices can be reduced to this example because of the invariance of Haar measure by unitary conjugation. Let $l \sim \mathrm{Unif}\{1, \dots, \frac{N}{2}\}$ be the number of systems to be measured. For $\mathbf{w} \in \{0,1\}^l$, define $M_{\mathbf{w}} = \bigotimes_{t=1}^{l}|w_t\rangle\langle w_t|$. We can write the post-measurement state as follows:

$$\rho_{l,\mathbf{w}}^{A_1\cdots A_k} = \frac{\int \mathrm{dHaar}(\varphi)\ \langle\varphi|^{\otimes l}M_{\mathbf{w}}\ |\varphi\rangle^{\otimes l}|\varphi\rangle\langle\varphi|^{\otimes k}}{\int \mathrm{dHaar}(\phi)\ \langle\phi|^{\otimes l}M_{\mathbf{w}}|\phi\rangle^{\otimes l}}.$$

The measurement channel related to the POVM $\mathcal{M} = \{|0\rangle\langle 0|, |1\rangle\langle 1|\}$ is :

$$\Lambda(\rho) = \langle 0|\rho|0\rangle|0\rangle\langle 0| + \langle 1|\rho|1\rangle|1\rangle\langle 1|.$$

If we apply the channel $\mathrm{id}\otimes\Lambda_2\otimes\cdots\otimes\Lambda_k = \mathrm{id}\otimes\Lambda\otimes\cdots\otimes\Lambda$ to the post-measurement state $\rho_{\mathbf{w}}^{A_1\cdots A_k}$

we obtain by writing $|\phi\rangle = \begin{pmatrix}\alpha_0\\ \alpha_1\end{pmatrix}$ and $p = |\alpha_1|^2$:

$$\begin{aligned}
&\mathrm{id}\otimes\Lambda\otimes\cdots\otimes\Lambda(\rho_{\mathbf{w}}^{A_1\cdots A_k})\\
&= \frac{\int \mathrm{dHaar}(\varphi)\langle\varphi|^{\otimes l}M_{\mathbf{w}}|\varphi\rangle^{\otimes l}|\varphi\rangle\langle\varphi|\otimes\Lambda(|\varphi\rangle\langle\varphi|)^{\otimes k-1}}{\int \mathrm{dHaar}(\phi)\langle\phi|^{\otimes l}M_{\mathbf{w}}|\phi\rangle^{\otimes l}}\\
&= \frac{\int \mathrm{dHaar}(\varphi)(|\alpha_0|^2)^{l-|\mathbf{w}|}(|\alpha_1|^2)^{|\mathbf{w}|}\begin{pmatrix}|\alpha_0|^2 & \alpha_0\bar{\alpha}_1\\ \alpha_1\bar{\alpha}_0 & |\alpha_1|^2\end{pmatrix}\otimes\begin{pmatrix}|\alpha_0|^2 & 0\\ 0 & |\alpha_1|^2\end{pmatrix}^{\otimes k-1}}{\int \mathrm{dHaar}(\varphi)(|\alpha_0|^2)^{l-|\mathbf{w}|}(|\alpha_1|^2)^{|\mathbf{w}|}}\\
&= \frac{\int_0^1 \mathrm{d}p\int_0^{2\pi}\frac{1}{2\pi}\,\mathrm{d}\theta(1-p)^{l-|\mathbf{w}|}p^{|\mathbf{w}|}\begin{pmatrix}1-p & \sqrt{p(1-p)}e^{\mathrm{i}\theta}\\ \sqrt{p(1-p)}e^{-\mathrm{i}\theta} & p\end{pmatrix}\otimes\begin{pmatrix}1-p & 0\\ 0 & p\end{pmatrix}^{\otimes k-1}}{\int_0^1 \mathrm{d}p\int_0^{2\pi}\frac{1}{2\pi}\,\mathrm{d}\theta(1-p)^{l-|\mathbf{w}|}p^{|\mathbf{w}|}}\\
&= \frac{\int_0^1 \mathrm{d}p(1-p)^{l-|\mathbf{w}|}p^{|\mathbf{w}|}\begin{pmatrix}1-p & 0\\ 0 & p\end{pmatrix}^{\otimes k}}{\int_0^1 \mathrm{d}p(1-p)^{l-|\mathbf{w}|}p^{|\mathbf{w}|}}\\
&= \int_0^1 \mathrm{d}p\;(l+1)\binom{l}{|\mathbf{w}|}(1-p)^{l-|\mathbf{w}|}p^{|\mathbf{w}|}\begin{pmatrix}1-p & 0\\ 0 & p\end{pmatrix}^{\otimes k}.
\end{aligned}$$

By tracing out all but the first system, we obtain an expression of the reduced post-measurement state:

$$\rho_{\mathbf{w}}^{A_1} = \int_0^1 \mathrm{d}p\;(l+1)\binom{l}{|\mathbf{w}|}(1-p)^{l-|\mathbf{w}|}p^{|\mathbf{w}|}\begin{pmatrix}1-p & 0\\ 0 & p\end{pmatrix} = \begin{pmatrix}1-\frac{|\mathbf{w}|+1}{l+2} & 0\\ 0 & \frac{|\mathbf{w}|+1}{l+2}\end{pmatrix}.$$

Hence if we denote by $p_\star = \frac{|\mathbf{w}|+1}{l+2}$ we have:

$$\begin{aligned}
&\left\|\mathrm{id}\otimes\Lambda\otimes\cdots\otimes\Lambda(\rho_{\mathbf{w}}^{A_1\cdots A_k}) - \mathrm{id}\otimes\Lambda\otimes\cdots\otimes\Lambda\left((\rho_{\mathbf{w}}^{A_1})^{\otimes k}\right)\right\|_1\\
&= \left\|\int_0^1 \mathrm{d}p\;(l+1)\binom{l}{|\mathbf{w}|}(1-p)^{l-|\mathbf{w}|}p^{|\mathbf{w}|}\left[\begin{pmatrix}1-p & 0\\ 0 & p\end{pmatrix}^{\otimes k} - \begin{pmatrix}1-p_\star & 0\\ 0 & p_\star\end{pmatrix}^{\otimes k}\right]\right\|_1.
\end{aligned}$$

By Stirling's approximation we have:

$$\binom{l}{|\mathbf{w}|}(1-p)^{l-|\mathbf{w}|}p^{|\mathbf{w}|} \le \frac{1}{\sqrt{2\pi|\mathbf{w}|(1-|\mathbf{w}|/l)}}\exp\left(-l\,\mathrm{KL}\left(\tfrac{|\mathbf{w}|}{l}\|p\right)\right),$$

where KL is the Kullback-Leibler divergence defined as $\mathrm{KL}(a\|b) = a\log(\frac{a}{b}) + (1-a)\log(\frac{1-a}{1-b})$ for $a, b \in (0,1)$ [1]. Hence if $S_\varepsilon = \left\{p\in[0,1] : \mathrm{KL}\left(\frac{|\mathbf{w}|}{l}\|p\right) > \varepsilon\right\}$ we have by the triangle inequality:

$$\begin{aligned}
&\left\|\int_{S_\varepsilon} \mathrm{d}p\;(l+1)\binom{l}{|\mathbf{w}|}(1-p)^{l-|\mathbf{w}|}p^{|\mathbf{w}|}\left[\begin{pmatrix}1-p & 0\\ 0 & p\end{pmatrix}^{\otimes k} - \begin{pmatrix}1-p_\star & 0\\ 0 & p_\star\end{pmatrix}^{\otimes k}\right]\right\|_1\\
&\le 2\int_{S_\varepsilon}\mathrm{d}p\;\frac{(l+1)}{\sqrt{2\pi|\mathbf{w}|(1-|\mathbf{w}|/l)}}\exp\left(-l\,\mathrm{KL}\left(\tfrac{|\mathbf{w}|}{l}\|p\right)\right) \le \frac{2(l+1)e^{-l\varepsilon}}{\sqrt{2\pi|\mathbf{w}|(1-|\mathbf{w}|/l)}}.
\end{aligned}$$

On the other hand, we have by the triangle and Pinsker's inequalities [1]:

$$
\begin{aligned}
&\left\|\int_{S_\varepsilon^c} \mathrm{d}p\ (l+1)\binom{l}{|\mathbf{w}|}(1-p)^{l-|\mathbf{w}|}p^{|\mathbf{w}|}\left[\begin{pmatrix}1-p & 0\\ 0 & p\end{pmatrix}^{\otimes k} - \begin{pmatrix}1-p_\star & 0\\ 0 & p_\star\end{pmatrix}^{\otimes k}\right]\right\|_1 \\
&\le \int_{S_\varepsilon^c} \mathrm{d}p\ (l+1)\binom{l}{|\mathbf{w}|}(1-p)^{l-|\mathbf{w}|}p^{|\mathbf{w}|}\left\|\begin{pmatrix}1-p & 0\\ 0 & p\end{pmatrix}^{\otimes k} - \begin{pmatrix}1-p_\star & 0\\ 0 & p_\star\end{pmatrix}^{\otimes k}\right\|_1 \\
&\le \int_{S_\varepsilon^c} \mathrm{d}p\ (l+1)\binom{l}{|\mathbf{w}|}(1-p)^{l-|\mathbf{w}|}p^{|\mathbf{w}|}\left\|\begin{pmatrix}1-p & 0\\ 0 & p\end{pmatrix}^{\otimes k} - \begin{pmatrix}1-|\mathbf{w}|/l & 0\\ 0 & |\mathbf{w}|/l\end{pmatrix}^{\otimes k}\right\|_1 \\
&\quad + \int_{S_\varepsilon^c} \mathrm{d}p\ (l+1)\binom{l}{|\mathbf{w}|}(1-p)^{l-|\mathbf{w}|}p^{|\mathbf{w}|}\left\|\begin{pmatrix}1-|\mathbf{w}|/l & 0\\ 0 & |\mathbf{w}|/l\end{pmatrix}^{\otimes k} - \begin{pmatrix}1-p_\star & 0\\ 0 & p_\star\end{pmatrix}^{\otimes k}\right\|_1 \\
&\le \int_{S_\varepsilon^c} \mathrm{d}p\ (l+1)\binom{l}{|\mathbf{w}|}(1-p)^{l-|\mathbf{w}|}p^{|\mathbf{w}|}\sqrt{2D\left(\begin{pmatrix}1-|\mathbf{w}|/l & 0\\ 0 & |\mathbf{w}|/l\end{pmatrix}^{\otimes k}\middle\|\begin{pmatrix}1-p & 0\\ 0 & p\end{pmatrix}^{\otimes k}\right)} \\
&\quad + \sqrt{2D\left(\begin{pmatrix}1-|\mathbf{w}|/l & 0\\ 0 & |\mathbf{w}|/l\end{pmatrix}^{\otimes k}\middle\|\begin{pmatrix}1-p_\star & 0\\ 0 & p_\star\end{pmatrix}^{\otimes k}\right)} \\
&= \int_{S_\varepsilon^c} \mathrm{d}p\ (l+1)\binom{l}{|\mathbf{w}|}(1-p)^{l-|\mathbf{w}|}p^{|\mathbf{w}|}\sqrt{2k\,\mathrm{KL}\left(\tfrac{|\mathbf{w}|}{l}\|p\right)} + \sqrt{2k\,\mathrm{KL}\left(\tfrac{|\mathbf{w}|}{l}\|p_\star\right)} \\
&\le \sqrt{2k\varepsilon} + \sqrt{2k\,\mathrm{KL}\left(\tfrac{|\mathbf{w}|}{l}\middle\|\tfrac{|\mathbf{w}|+1}{l+2}\right)} \le \sqrt{2k\varepsilon} + \sqrt{\frac{4k}{l}}.
\end{aligned}
$$

Therefore by the triangle inequality we deduce that:

$$
\begin{aligned}
&\left\|\mathrm{id}\otimes\Lambda\otimes\cdots\otimes\Lambda(\rho_{\mathbf{w}}^{A_1\cdots A_k}) - \mathrm{id}\otimes\Lambda\otimes\cdots\otimes\Lambda\left((\rho_{\mathbf{w}}^{A_1})^{\otimes k}\right)\right\|_1 \\
&= \left\|\int_0^1 \mathrm{d}p\ (l+1)\binom{l}{|\mathbf{w}|}(1-p)^{l-|\mathbf{w}|}p^{|\mathbf{w}|}\left[\begin{pmatrix}1-p & 0\\ 0 & p\end{pmatrix}^{\otimes k} - \begin{pmatrix}1-p_\star & 0\\ 0 & p_\star\end{pmatrix}^{\otimes k}\right]\right\|_1 \\
&\le \frac{2(l+1)e^{-l\varepsilon}}{\sqrt{2\pi|\mathbf{w}|(1-|\mathbf{w}|/l)}} + \sqrt{2k\varepsilon} + \sqrt{\frac{4k}{l}} \\
&\le \frac{2}{(l+1)\sqrt{2\pi|\mathbf{w}|(1-|\mathbf{w}|/l)}} + \sqrt{\frac{2k\log\left((l+1)^2\right)}{l}} + \sqrt{\frac{4k}{l}} \le \sqrt{\frac{9k\log(l+1)}{l}}
\end{aligned}
$$

where the last inequalities are achieved for $\varepsilon = \frac{2}{l}\log(l+1)$. Now if we take the average under $l \sim \mathrm{Unif}\{1,\dots,\frac{N}{2}\}$:

$$
\begin{aligned}
\mathbb{E}_l\left[\left\|\mathrm{id}\otimes\Lambda^{\otimes k-1}(\rho_{l,\mathbf{w}}^{A_1\cdots A_k}) - \mathrm{id}\otimes\Lambda^{\otimes k-1}\left((\rho_{l,\mathbf{w}}^{A_1})^{\otimes k}\right)\right\|_1^2\right] &\le \frac{2}{N}\sum_{l=1}^{N/2}\frac{9k\log(l+1)}{l} \\
&\le \frac{18k\log^2(N)}{N}.
\end{aligned}
$$

Finally by Cauchy Schwarz's inequality

$$
\mathbb{E}_l\left[\left\|\mathrm{id}\otimes\Lambda^{\otimes k-1}(\rho_{l,\mathbf{w}}^{A_1\cdots A_k}) - \mathrm{id}\otimes\Lambda^{\otimes k-1}\left((\rho_{l,\mathbf{w}}^{A_1})^{\otimes k}\right)\right\|_1\right] \le \sqrt{\frac{18k\log^2(N)}{N}}.
$$

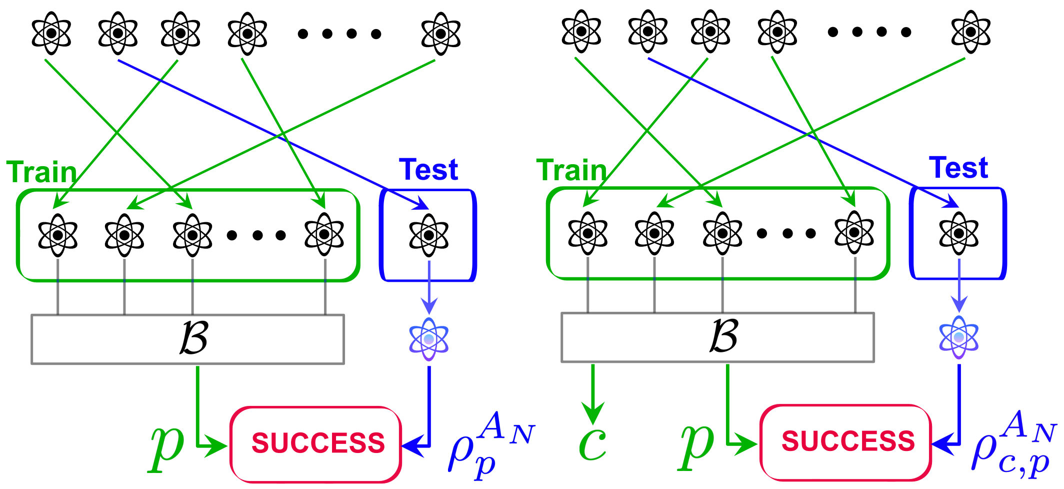

Supplementary Figure 1: A general algorithm for learning properties of quantum states in the non-i.i.d. setting. Left (resp. Right) the algorithm $\mathcal{B}$ is not (resp. is) allowed to output calibration information c. Success occurs if prediction p is (approximately) compatible with the remaining post-measurement test copies $\rho_p^{A_N}$ or $\rho_{c,p}^{A_N}$.

Supplementary Note 3 – Generalizing the i.i.d. setting without calibration information

A potential objection to Algorithm 1 of the main text might be that it produces more information than strictly required. Indeed, in addition to the prediction provided by $\mathcal{A}\left(\rho_{\mathbf{w}}^{A_1\cdots A_k}\right)$, Algorithm 1 of the main text also furnishes the observations $\mathbf{w}$ that lead to the given prediction. These observations hold calibration-related data, intended for future utilization. Notably, they are not essential for the immediate prediction task at hand. In this section, we introduce a framework for extending non-adaptive algorithms to the non-i.i.d. setting without returning calibration information. This extension is achievable for a broad range of problems that can be defined by a function under reasonable assumptions. Instead of Definition 6 of the main text for the error probability with calibration, we will use the following definition of the error probability without calibration (See Supplementary Figure 1 for an illustration of algorithms without and with calibration information.):

Supplementary Definition 1 (Error probability in the non-i.i.d. setting (no calibration data)). Let $N \geq 1$ be a positive integer, $A_1 \cong A_2 \cong \cdots \cong A_N$ be N isomorphic quantum systems. Let $\rho^{A_1\cdots A_N} \in \mathrm{D}(A_1\cdots A_N)$. A learning algorithm $\mathcal{B} : \mathrm{L}(A_1 \dots A_{N-1}) \to \mathbb{C}^{\mathcal{P}}$ has error probability on ρ given by:

$$\delta_{\mathcal{B}}(N, \rho^{A_1\cdots A_N}, \varepsilon) = \mathbb{P}_{p\sim\mathcal{B}(\rho)}\left[\left(p, \rho_p^{A_N}\right) \notin \mathrm{SUCCESS}_\varepsilon\right],$$

where p follows the probability measure $\mathcal{B}(\rho^{A_1\dots A_{N-1}})$ and we recall that $\rho_p^{A_N}$ is defined by conditioning on the outcome p of the measurement $\mathcal{B}$ on the systems $A_1 \dots A_{N-1}$ of ρ.

Note that, if ρ is i.i.d., the conditioning on p does not have any effect on the post-measurement state and Supplementary Definition 1 coincides with the usual definition of the error probability. The following example illustrates the possible difference that conditioning on calibration data has.

Supplementary Example 2. We denote the weight of an element $\mathbf{x} \in \{0,1\}^n$ by $|\mathbf{x}| = \sum_{i=1}^n x_i$. Let $\rho^{A_1\cdots A_N} = \sum_{\mathbf{x}\in\{0,1\}^n} \frac{1}{2^n}|\mathbf{x}\rangle\langle\mathbf{x}|^{\otimes N}$ be a permutation invariant state. We want to predict the (average) weight of the state. A possible algorithm $\mathcal{B}$ is to measure the first system A_1 with the canonical basis

$\{|\mathbf{x}\rangle\langle\mathbf{x}|\}_{\mathbf{x}\in\{0,1\}^n}$, observe $\mathbf{x} \in \{0,1\}^n$ and return the prediction $p = |\mathbf{x}|$ and possibly the calibration $c = \mathbf{x}$. The post-measurement state conditioned on the prediction-related information p is:

$$\rho_p^{A_N} = \frac{1}{\binom{n}{p}} \sum_{\mathbf{y}\in\{0,1\}^n:|\mathbf{y}|=p} |\mathbf{y}\rangle\langle\mathbf{y}|.$$

On the other hand, the post-measurement state conditioned on the prediction and calibration information is:

$$\rho_{c,p}^{A_N} = |\mathbf{x}\rangle\langle\mathbf{x}|.$$

The states $\rho_p^{A_N}$ and $\rho_{c,p}^{A_N}$ are in general different. Infact, we have, with probability at least $1 - 1/2^{n-1}$, $\left\|\rho_p^{A_N} - \rho_{c,p}^{A_N}\right\|_1 = 2\left(1 - 1/\binom{n}{p}\right) \geq 2 - 2/n$.

The learning problems we consider in this section are defined by a function under reasonable assumptions.

Supplementary Definition 2. Consider a function $\mathtt{d}$ designed to determine a particular property concerning quantum states. The problem of learning the property of quantum states can be formulated using the following SUCCESS set:

$$\mathrm{SUCCESS}_\varepsilon = \{(p,\sigma) : \mathtt{d}(p,\sigma) \leq \varepsilon\} \subset \mathcal{P} \times \mathrm{D}(A)$$

where $\mathcal{P}$ is a set. The function $\mathtt{d}$ should satisfy the following properties:

(a) **Non-negativity:** for all (p,σ), $\mathtt{d}(p,\sigma) \geq 0$.

(b) **Boundedness:** there is a constant $C > 0$ such that for all (p,σ), $\mathtt{d}(p,\sigma) \leq C$.

(c) **Robustness:** for all (p,σ) and (p,ρ), $|\mathtt{d}(p,\sigma) - \mathtt{d}(p,\rho)| \leq \frac{1}{2}\|\sigma - \rho\|_1$.

(d) **Convexity in the second entry:** for all $\alpha \in (0,1)$, (p,σ) and (p,ρ),

$$\alpha\mathtt{d}(p,\sigma) + (1-\alpha)\mathtt{d}(p,\rho) \geq \mathtt{d}\left(p, \alpha\sigma + (1-\alpha)\rho\right).$$

Many problems about learning properties of quantum states can be formulated using Supplementary Definition 2.

Supplementary Example 3 (State tomography). The problem of state tomography corresponds to the trace distance function $\mathtt{d} = \mathtt{d}_{\mathrm{Tr}}$ where the trace distance is defined by $\mathtt{d}_{\mathrm{Tr}}(\rho,\sigma) = \frac{1}{2}\|\rho - \sigma\|_1$. The trace distance satisfies all the conditions in Supplementary Definition 2 for $C = \frac{1}{2}$.

Supplementary Example 4 (Shadow tomography). Here we consider M observables $0 \preccurlyeq O_1, \ldots, O_M \preccurlyeq \mathbb{I}$. The shadow tomography problem corresponds to the function $\mathtt{d}$ between the tuple $\mathbf{p} = (\mu_1, \ldots, \mu_M) \in [0,1]^M$ and the state σ defined as follows:

$$\mathtt{d}(\mathbf{p},\sigma) = \max_{1\leq i\leq M} |\mu_i - \mathrm{Tr}\left[O_i\sigma\right]|.$$

Clearly, $0 \leq \mathtt{d}(\mathbf{p},\sigma) \leq 2$. Now, let σ, ρ be two states and $\mathbf{p} = (\mu_1, \ldots, \mu_M)$ be a tuple, we have:

$$\begin{aligned}
|\mathtt{d}(\mathbf{p},\sigma) - \mathtt{d}(\mathbf{p},\rho)| &= \left|\max_{1\leq i\leq M} |\mu_i - \mathrm{Tr}\left[O_i\sigma\right] - \max_{1\leq i\leq M} |\mu_i - \mathrm{Tr}\left[O_i\rho\right]\right| \\
&\leq \max_{1\leq i\leq M} ||\mu_i - \mathrm{Tr}\left[O_i\sigma\right] - |\mu_i - \mathrm{Tr}\left[O_i\rho\right]| \\
&\leq \max_{1\leq i\leq M} |\mathrm{Tr}\left[O_i\sigma\right] - \mathrm{Tr}\left[O_i\rho\right]| \leq \frac{1}{2}\|\sigma - \rho\|_1.
\end{aligned}$$

Moreover for $\alpha \in [0,1]$:

$$\begin{aligned}\alpha \mathtt{d}(\mathbf{p},\sigma) + (1-\alpha)\mathtt{d}(\mathbf{p},\rho) &= \alpha \max_{1\le i\le M} |\mu_i - \mathrm{Tr}\left[O_i\sigma\right]| + (1-\alpha) \max_{1\le i\le M} |\mu_i - \mathrm{Tr}\left[O_i\rho\right]| \\ &\ge \max_{1\le i\le M} \left(\alpha\left|\mu_i - \mathrm{Tr}\left[O_i\sigma\right]\right| + (1-\alpha)\left|\mu_i - \mathrm{Tr}\left[O_i\rho\right]\right|\right) \\ &\ge \max_{1\le i\le M} \left|\mu_i - \alpha\mathrm{Tr}\left[O_i\sigma\right] - (1-\alpha)\mathrm{Tr}\left[O_i\rho\right]\right| \\ &= \mathtt{d}\left(\mathbf{p}, \alpha\sigma + (1-\alpha)\rho\right).\end{aligned}$$

Finally $\mathtt{d}$ satisfies the conditions in Supplementary Definition 2.

Supplementary Example 5 (Verification of a pure state). Given an ideal pure state $|\Psi\rangle$ and a binary prediction $p \in \{0,1\}$ representing whether the algorithm accepts or rejects, we define $\mathtt{d}$ as follows:

$$\mathtt{d}(p,\sigma) = p + (1-p)(1 - \langle\Psi|\sigma|\Psi\rangle).$$

If we can prove that:

$$\mathbb{P}_p\left[\mathtt{d}(p,\rho_p^{A_N}) > \varepsilon\right] \le \delta$$

Then we have both completeness and soundness:

- **Completeness.** If the verifier receives the state $\rho = |\Psi\rangle\langle\Psi|^{\otimes N}$, then $\langle\Psi|\rho_p^{A_N}|\Psi\rangle = \langle\Psi||\Psi\rangle\langle\Psi||\Psi\rangle = 1$. On the other hand, with probability $1-\delta$, we have $p = p + (1-p)(1 - \langle\Psi|\rho_p^{A_N}|\Psi\rangle) \le \varepsilon$ hence $p = 0$ and the verifier accepts.
- **Soundness.** If the verifier accepts, i.e., $p = 0$, then we have with a probability at least $1-\delta$, $1 - \langle\Psi|\rho_p^{A_N}|\Psi\rangle = p + (1-p)(1 - \langle\Psi|\rho_p^{A_N}|\Psi\rangle) \le \varepsilon$ therefore the post-measurement state $\rho_p^{A_N}$ satisfies $\mathrm{Tr}\left[|\Psi\rangle\langle\Psi|\rho_p^{A_N}\right] \ge 1-\varepsilon$ with a probability at least $1-\delta$.

Let us show that $\mathtt{d}$ satisfies the conditions in Supplementary Definition 2. First, $\mathtt{d}$ is clearly non negative and at most 1. For two states σ and τ, we have:

$$|\mathtt{d}(p,\tau) - \mathtt{d}(p,\sigma)| = (1-p)|\langle\Psi|\sigma-\tau|\Psi\rangle| \le \frac{1}{2}\|\sigma-\tau\|_1$$

so $\mathtt{d}$ satisfies the robustness condition. For the convexity, let $\alpha \in [0,1]$, we have:

$$\begin{aligned}\alpha\mathtt{d}(p,\sigma) + (1-\alpha)\mathtt{d}(p,\tau) &= p + \alpha(1-p)(1-\langle\Psi|\sigma|\Psi\rangle) + (1-\alpha)(1-p)(1-\langle\Psi|\tau|\Psi\rangle) \\ &= p + (1-p)\left(1 - \langle\Psi|\alpha\sigma + (1-\alpha)\tau|\Psi\rangle\right) \\ &= \mathtt{d}\left(p, \alpha\sigma + (1-\alpha)\tau\right).\end{aligned}$$

Supplementary Example 6 (Testing mixedness of states). In the problem of testing mixedness of states, we would like to test whether $\sigma = \frac{\mathbb{I}}{d}$ or $\frac{1}{2}\left\|\sigma - \frac{\mathbb{I}}{d}\right\|_1 > \varepsilon$. We can define $\mathtt{d}$ for a binary prediction $p \in \{0,1\}$ and a quantum state σ:

$$\mathtt{d}(p,\sigma) = p + \frac{1}{2}(1-p)\left\|\sigma - \tfrac{\mathbb{I}}{d}\right\|_1$$

where $p \in \{0,1\}$ represents the outcome of the algorithm, 0 for the null hypothesis and 1 for the alternate hypothesis. Similar to the previous example, $\mathtt{d}$ satisfies the conditions in Supplementary Definition 2.

Moreover, we can show that $\mathtt{d}(p,\sigma) \le \varepsilon$ implies that algorithm $\mathcal{B}$ is correct. Indeed, if $\sigma = \frac{\mathbb{I}}{d}$ then $\mathtt{d}(p,\sigma) = p$ thus $p = \mathtt{d}(p,\sigma) \le \varepsilon$ implying $p = 0$. On the other hand, if $\frac{1}{2}\left\|\sigma - \frac{\mathbb{I}}{d}\right\|_1 > \varepsilon$ then we have $(1-p)\varepsilon < \frac{1}{2}(1-p)\left\|\sigma - \frac{\mathbb{I}}{d}\right\|_1 \le \mathtt{d}(p,\sigma) \le \varepsilon$ implying $(1-p) < 1$ and finally $p = 1$.

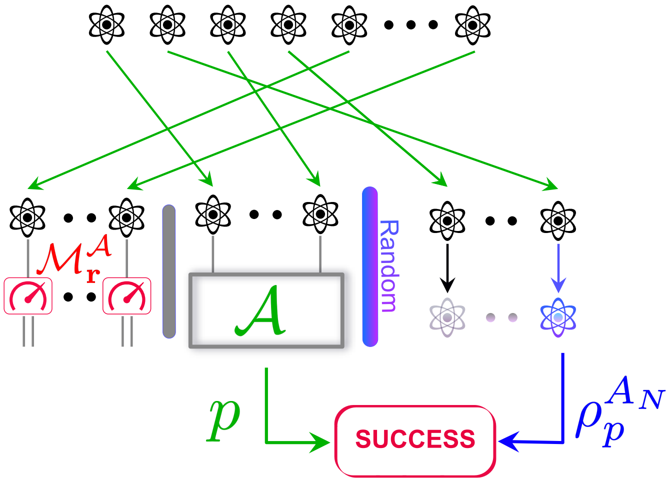

Supplementary Figure 2: Illustration of Supplementary Algorithm 1. Supplementary Algorithm 1 measures a large number of the state's subsystems using $\mathcal{M}_{\mathbf{r}}^{\mathcal{A}}$ that represents measurement devices uniformly chosen from the i.i.d. algorithm's set of POVMs $\{\mathcal{M}_t\}_t$ (red and green parts). Then, Supplementary Algorithm 1 applies the data processing of Algorithm $\mathcal{A}$ to the outcomes of a part of these subsystems (green part) leading to a prediction p. Success occurs if p is (approximately) compatible with the remaining post-measurement test copy $\rho_p^{A_N}$.

Supplementary Algorithm 1 Predicting properties of quantum states in the non-i.i.d. setting without calibration information - Non-adaptive algorithms

---

Require: The measurements $\{\mathcal{M}_t^{\mathcal{A}}\}_{1\le t\le k_{\mathcal{A}}}$ of algorithm $\mathcal{A}$.
  A permutation invariant state $\rho^{A_1\cdots A_N}$.
Ensure: Adapt the algorithm $\mathcal{A}$ to non-i.i.d. inputs $\rho^{A_1\cdots A_N}$.
  Sample $l \sim \mathrm{Unif}\{k+1,\dots,k+\frac{N}{2}\}$ and $(r_1,\dots,r_l) \overset{iid}{\sim} \mathrm{Unif}\{1,\dots,k_{\mathcal{A}}\}$
  For $t=k+1,\dots,l$, apply $\mathcal{M}_{r_t}^{\mathcal{A}}$ to system A_t and obtain outcome $\mathbf{w} \leftarrow \bigotimes_{t=k+1}^{l} \mathcal{M}_{r_t}^{\mathcal{A}}(\rho)$
  For $t=1,\dots,k$, apply $\mathcal{M}_{r_t}^{\mathcal{A}}$ to system A_t and obtain outcome $\mathbf{v} \leftarrow \bigotimes_{t=1}^{k} \mathcal{M}_{r_t}^{\mathcal{A}}(\rho_{\mathbf{w}})$
  For $t=1,\dots,k_{\mathcal{A}}$, let $s(t)\in[k]$ be the first integer such that $r_{s(t)}=t$
  Run the prediction of algorithm $\mathcal{A}$ to the measurement outcomes $v_{s(1)},\dots,v_{s(k_{\mathcal{A}})}$ and obtain p
  return p.

For problems defined with a function $\mathtt{d}$ satisfying the conditions in Supplementary Definition 2, we propose Supplementary Algorithm 1 in the non-i.i.d. setting (See Supplementary Figure 2 for an illustration).

Recall the definition of the error probability for algorithms without calibration information:

$$\delta_{\mathcal{B}}(N,\rho^{A_1\cdots A_N},\varepsilon) = \mathbb{P}_p\left[\left(p,\rho_p^{A_N}\right)\notin \mathrm{SUCCESS}_{\varepsilon}\right].$$

The main result of this section is to control the error probability of Supplementary Algorithm 1. Recall that we consider problems defined by a function $\mathtt{d}$ upper bounded by a constant $C>0$ (see Supplementary Definition 2) and $\rho_{l,\mathbf{r},\mathbf{w}}^{A_N} = \mathrm{Tr}_{-A_N}\left[\rho_{l,\mathbf{r},\mathbf{w}}^{A_{l+1}\cdots A_N}\right]$.

Supplementary Theorem 1. Let $\varepsilon>0$ and $k_{\mathcal{A}}<k<N/2$. Let $\mathcal{A}$ be a non-adaptive algorithm performing incoherent measurements with $\{\mathcal{M}_t\}_{1\le t\le k_{\mathcal{A}}}$. There is an algorithm (without calibration information) $\mathcal{B}$ suitable for arbitrary input states, performing i.i.d. measurements drawn from

$\mathrm{Unif}\{\mathcal{M}_t\}_{1\le t\le k_{\mathcal{A}}}$ and possessing an error probability satisfying for all $\eta > 0$:

$$\delta_{\mathcal{B}}(N, \rho^{A_1\cdots A_N}, \varepsilon)$$
$$\le \frac{C}{\varepsilon}\sup_{l,\mathbf{r},\mathbf{w}} \delta_{\mathcal{A}}\left(k_{\mathcal{A}}, \left(\rho^{A_N}_{l,\mathbf{r},\mathbf{w}}\right)^{\otimes k_{\mathcal{A}}}, \eta\right) + \frac{2\eta}{\varepsilon} + \frac{C}{\varepsilon}k_{\mathcal{A}}e^{-k/k_{\mathcal{A}}} + \frac{4C}{\varepsilon}\sqrt{\frac{k^2\log(d)}{N\eta^2}} + \frac{2C}{\varepsilon}\sqrt{\frac{k^2\log(d)}{N}}.$$

Proof Here, we show how to relate the approximation of the post-measurement state $\rho^{A_N}_p$ with the approximation of the post-measurement state $\rho^{A_N}_{l,\mathbf{r},\mathbf{w},p}$.

Supplementary Lemma 1. We have for all $\eta > 0$:

$$\mathbb{P}_p\left[(p, \rho^{A_N}_p) \notin \mathrm{SUCCESS}_\varepsilon\right] \le \frac{\eta}{\varepsilon} + \frac{C}{\varepsilon}\,\mathbb{P}_{l,\mathbf{r},\mathbf{w},p}\left[(p, \rho^{A_N}_{l,\mathbf{r},\mathbf{w},p}) \notin \mathrm{SUCCESS}_\eta\right].$$

Once we have this lemma, we obtain the theorem by applying Theorem 5 of the main text.

Proof We use the notation $\bigotimes_{t=1}^{l}\mathcal{M}_t = \{M_{\mathbf{x}}\}_{\mathbf{x}}$. Since $\mathtt{d}$ is convex in the second entry, we have:

$$\begin{aligned}
\mathbb{E}_{l,\mathbf{r},\mathbf{w},p}\left[\mathtt{d}\left(p, \rho^{A_N}_{l,\mathbf{r},\mathbf{w},p}\right)\right] &= \mathbb{E}_{l,\mathbf{r}}\left[\sum_{\mathbf{x}} \mathrm{Tr}\left[M_{\mathbf{x}}\rho\right]\mathtt{d}\left(p, \rho^{A_N}_{l,\boldsymbol{r},\boldsymbol{x},p}\right)\right]\\
&= \mathbb{E}_{l,\mathbf{r}}\left[\sum_{y}\sum_{\mathbf{x}:p=y} \mathrm{Tr}\left[M_{\mathbf{x}}\rho\right]\mathtt{d}\left(y, \rho^{A_N}_{l,\boldsymbol{r},\boldsymbol{x},y}\right)\right]\\
&\ge \mathbb{E}_{l,\mathbf{r}}\left[\sum_{y} q(y)\mathtt{d}\left(y, \frac{1}{q(y)}\sum_{\mathbf{x}:p=y} \mathrm{Tr}\left[M_{\mathbf{x}}\rho\right]\rho^{A_N}_{l,\boldsymbol{r},\boldsymbol{x},y}\right)\right]\\
&= \mathbb{E}_{l,\mathbf{r}}\left[\sum_{y} q(y)\mathtt{d}\left(y, \rho^{A_N}_{l,\mathbf{r},y}\right)\right] = \mathbb{E}_p\left[\mathtt{d}\left(p, \rho^{A_N}_p\right)\right]
\end{aligned}$$

where we use the notation $q(y) = \sum_{\mathbf{x}:p=y}\mathrm{Tr}\left[M_{\mathbf{x}}\rho\right]$. Finally, we apply a simple Markov's inequality then the last inequality to obtain for all $\eta > 0$:

$$\begin{aligned}
\mathbb{P}_p\left[\left(p, \rho^{A_N}_p\right) \notin \mathrm{SUCCESS}_\varepsilon\right] &= \mathbb{P}_p\left[\mathtt{d}\left(p, \rho^{A_N}_p\right) > \varepsilon\right]\\
&\le \frac{1}{\varepsilon}\mathbb{E}_p\left[\mathtt{d}\left(p, \rho^{A_N}_p\right)\right]\\
&\le \frac{1}{\varepsilon}\mathbb{E}_{l,\mathbf{r},\mathbf{w},p}\left[\mathtt{d}\left(p, \rho^{A_N}_{l,\mathbf{r},\mathbf{w},p}\right)\right]\\
&= \frac{1}{\varepsilon}\int_0^C \mathbb{P}_{l,\mathbf{r},\mathbf{w},p}\left[\mathtt{d}\left(p, \rho^{A_N}_{l,\mathbf{r},\mathbf{w},p}\right) \ge x\right]\mathrm{d}x\\
&\le \frac{\eta}{\varepsilon} + \frac{C}{\varepsilon}\,\mathbb{P}_{l,\mathbf{r},\mathbf{w},p}\left[(p, \rho^{A_N}_{l,\mathbf{r},\mathbf{w},p}) \notin \mathrm{SUCCESS}_\eta\right].
\end{aligned}$$

Similarly, using Supplementary Lemma 1 we can generalize Theorem 8 of the main text to control the error probability without calibration information of Supplementary Algorithm 2:

Supplementary Theorem 2. Let $\varepsilon > 0$ and $1 \le k < N/2$. Let $\mathcal{A}$ be a general algorithm. Supplementary Algorithm 2 (without calibration information) has an error probability satisfying for all $\eta > 0$:

$$\delta_{\mathcal{B}}\left(N, \rho^{A_1\cdots A_N}, \varepsilon\right) \le \frac{C}{\varepsilon}\sup_{l,\mathbf{w}} \delta_{\mathcal{A}}\left(k, (\rho^{A_N}_{l,\mathbf{w}})^{\otimes k}, \eta\right) + \frac{2\eta}{\varepsilon} + \frac{12C}{\varepsilon}\sqrt{\frac{2k^3d^2\log(d)}{N\eta^2}} + \frac{2C}{\varepsilon}\sqrt{\frac{2k^3d^2\log(d)}{N}}.$$

Supplementary Algorithm 2 Predicting properties of quantum states in the non-i.i.d. setting without calibration information - General algorithms

Require: Measurement $\mathcal{A} : \mathrm{L}(A_1 \dots A_k) \to \mathbb{C}^{\mathcal{P}}$. A permutation invariant state $\rho^{A_1 \cdots A_N}$.
Ensure: Adapt the algorithm $\mathcal{A}$ to non-i.i.d. inputs $\rho^{A_1 \cdots A_N}$.
Run algorithm $\mathcal{A}$ on systems $A_1 \dots A_k$ and obtain outcome $p \leftarrow \mathcal{A}(\rho)$
Sample $l \sim \mathrm{Unif}\{k+1, \dots, k+\frac{N}{2}\}$
Apply $\mathcal{M}_{\mathrm{dist}}$ to each system A_{k+1} to A_l and obtain outcome $\mathbf{w} \leftarrow \mathcal{M}_{\mathrm{dist}}^{\otimes(l-k)}(\rho)$
return p.

Supplementary Note 4 – Verification of pure states in expectation

In Section 4.4.2 of the main text, we mentioned that for the problems of verifying one pure state, the soundness condition is often formulated in terms of expectation rather than probability. We used the formulation with probability in Section 4.4.2 of the main text because we wanted to verify many pure states simultaneously. Here, we show that a similar statement can be formulated in expectation if we focus on verifying one pure state. The techniques are similar but do not follow directly from Theorem 5 nor Theorem 8 of the main text.

Recall that in the context of verifying a pure state, we are given an ideal known state Ψ and copies of an unknown state. The objective is to verify whether the received reduced state is exactly the ideal state or far from it in fidelity. Formally, a verifier should satisfy the completeness and soundness conditions:

1. ***Completeness.*** The verifier accepts upon receiving the pure i.i.d. states $|\Psi\rangle\langle\Psi|^{\otimes N}$ with high probability, i.e., if Π_{Accept} represents the observable in which the verification protocol accepts, the completeness condition is met when the following inequality holds:

$$\mathrm{Tr}\left[\Pi_{\mathrm{Accept}} |\Psi\rangle\langle\Psi|^{\otimes N-1}\right] \geq 1 - \varepsilon.$$

2. ***Soundness.*** When the verifier accepts, the quantum state passing the verification protocol (post-measurement state conditioned on a passing event) is close to the pure ideal state $|\Psi\rangle\langle\Psi|$ with high probability, i.e., if Π_{Accept} represents the observable in which the verification protocol accepts, the soundness condition is met when the following inequality holds:

$$\mathrm{Tr}\left[\Pi_{\mathrm{Accept}} \otimes (\mathbb{I} - |\Psi\rangle\langle\Psi|)\, \rho^{A_1 \cdots A_N}\right] \leq \varepsilon.$$

In the latter scenario, the protocol can receive a possibly highly entangled state $\rho^{A_1 \cdots A_N}$.

We can show the following proposition:

Supplementary Proposition 1. A pure state can be verified using Clifford measurements and a number of copies satisfying:

$$N = \frac{800^2 e^4 \log(5/\varepsilon)^2 \log(d)}{\varepsilon^6}.$$

Proof Let $|\Psi\rangle$ be the ideal state. We will use classical shadows with Clifford random measurements [2]. Let $K = 2\log(1/\varepsilon)$, $k = \frac{4e^2 5^2 \log(5/\varepsilon)}{\varepsilon^2}$, $N = \frac{8^2 k^2 \log(d)}{\varepsilon^2}$, $l \sim \mathrm{Unif}\{k+1, \dots, k+N/2\}$ and $U_1, \dots, U_l \sim \mathbf{Cl}(2^n)$. The state $\rho^{A_1 \cdots A_l}$ is measured with the corresponding basis of $U_1 \otimes \cdots \otimes U_l$ and the outcomes are denoted $(\mathbf{v}, \mathbf{w}) = (v_1, \dots, v_k, w_{k+1}, \dots, w_l)$. Then K classical shadows are constructed as follows:

$$\hat{\rho}_{(j)} = \frac{1}{N_0/K} \sum_{t=(j-1)k/K}^{jk/K} (d+1) U_t^\dagger |v_t\rangle\langle v_t| U_t - \mathbb{I} \qquad \text{for} \qquad 1 \leq j \leq K.$$

Next we use the median of means statistic for $\mathbf{v} = (v_1, \dots, v_k)$ [2]:

$$\mu_{\mathbf{v}} = \text{median}\left\{\text{Tr}\left[\hat{\rho}_{(j)}|\Psi\rangle\langle\Psi|\right]\right\}_{1\le j\le K}.$$

We define the observable corresponding to 'Accept':

$$\Pi_{\text{Accept}} = \frac{2}{N}\sum_{l=k+1}^{k+N/2}\mathbb{E}_{U\sim\mathbf{Cl}(2^n)}\left[\sum_{\mathbf{v},\mathbf{w}}\mathbf{1}\{\mu_{\mathbf{v}} \ge 1-\varepsilon/5\}M_{\mathbf{v}}\otimes M_{\mathbf{w}}\otimes\mathbb{I}\right]$$

where $M_{\mathbf{v}} = \bigotimes_{t=1}^{k} U_t|v_t\rangle\langle v_t|U_t^\dagger$ and $M_{\mathbf{w}} = \bigotimes_{t=k+1}^{l} U_t|w_t\rangle\langle w_t|U_t^\dagger$.

Completeness. When the verifier receives the i.i.d. state $\rho^{A_1\cdots A_N} = |\Psi\rangle\langle\Psi|^{\otimes N}$, it should accept with probability at least $1-\varepsilon$. The probability of acceptance can be expressed using the notation $\mathbb{E}_{(\mathbf{v},\mathbf{w})\sim\rho}[\cdot] = \sum_{\mathbf{v},\mathbf{w}}\text{Tr}\left[(M_{\mathbf{v}}\otimes M_{\mathbf{w}})\rho\right][\cdot]$:

$$\begin{aligned}\text{Tr}\left[\Pi_{\text{Accept}}|\Psi\rangle\langle\Psi|^{\otimes N}\right] &= \mathbb{E}_{U\sim\mathbf{Cl}(2^n)}\left[\frac{2}{N}\sum_{l=k+1}^{k+N/2}\sum_{\mathbf{v},\mathbf{w}}\mathbf{1}\{\mu_{\mathbf{v}}\ge 1-\varepsilon/5\}\text{Tr}\left[M_{\mathbf{v}}\otimes M_{\mathbf{w}}|\Psi\rangle\langle\Psi|^{\otimes l}\right]\right]\\ &= \mathbb{P}_{l,U\sim\mathbf{Cl}(2^n),(\mathbf{v},\mathbf{w})\sim|\Psi\rangle\langle\Psi|^{\otimes l}}\left[\mu_{\mathbf{v}}\ge 1-\varepsilon/5\right]\ge 1-\varepsilon\end{aligned}$$

where we use the fact that here the input state $|\Psi\rangle\langle\Psi|^{\otimes N}$ is i.i.d., the observable $O = |\Psi\rangle\langle\Psi|$ satisfies $\text{Tr}\left[O|\Psi\rangle\langle\Psi|\right] = 1$ and the correctness of classical shadow protocol ([2], $k = \frac{4e^2 5^2\log(5/\varepsilon)}{\varepsilon^2}$).

Soundness. By the randomized local de Finetti Theorem 4 of the main text we have using the notation $\mathbb{E}_{l,U,(\mathbf{v},\mathbf{w})\sim\rho}[X] = \frac{2}{N}\sum_{l=k+1}^{k+N/2}\mathbb{E}_{U\sim\mathbf{Cl}(2^n)}\left[\sum_{\mathbf{v},\mathbf{w}}\text{Tr}\left[(M_{\mathbf{v}}\otimes M_{\mathbf{w}})\rho\right]X\right]$:

$$\begin{aligned}&\text{Tr}\left[\Pi_{\text{Accept}}\otimes(\mathbb{I}-|\Psi\rangle\langle\Psi|)\rho^{A_1\cdots A_{N+1}}\right]\\ &= \mathbb{E}_{l,U,(\mathbf{v},\mathbf{w})\sim\rho}\left[\mathbf{1}\{\mu_{\mathbf{v}}\ge 1-\varepsilon/5\}\text{Tr}\left[(\mathbb{I}-|\Psi\rangle\langle\Psi|)\rho^{A_N}_{\mathbf{v},\mathbf{w}}\right]\right]\\ &\le \mathbb{E}_{l,U,(\mathbf{v},\mathbf{w})\sim\rho}\left[\mathbf{1}\{\mu_{\mathbf{v}}\ge 1-\varepsilon/5\}\|\rho^{A_N}_{\mathbf{w}}-\rho^{A_N}_{\mathbf{v},\mathbf{w}}\|_1\right] + \mathbb{E}_{l,U,(\mathbf{v},\mathbf{w})\sim\rho}\left[\mathbf{1}\{\mu_{\mathbf{v}}\ge 1-\varepsilon/5\}\text{Tr}\left[(\mathbb{I}-|\Psi\rangle\langle\Psi|)\rho^{A_N}_{\mathbf{w}}\right]\right]\\ &= \mathbb{E}_{l,U,(\mathbf{v},\mathbf{w})\sim\rho}\left[\mathbf{1}\{\mu_{\mathbf{v}}\ge 1-\varepsilon/5\}\|\rho^{A_N}_{\mathbf{w}}-\rho^{A_N}_{\mathbf{v},\mathbf{w}}\|_1\right]\\ &\quad + \mathbb{E}_{l,U,(\mathbf{v},\mathbf{w})\sim\rho}\left[\mathbf{1}\{\mu_{\mathbf{v}}\ge 1-\varepsilon/5\}\mathbf{1}\{\text{Tr}\left[(\mathbb{I}-|\Psi\rangle\langle\Psi|)\rho^{A_N}_{\mathbf{w}}\right]\le 2\varepsilon/5\}\text{Tr}\left[(\mathbb{I}-|\Psi\rangle\langle\Psi|)\rho^{A_N}_{\mathbf{w}}\right]\right]\\ &\quad + \mathbb{E}_{l,U,(\mathbf{v},\mathbf{w})\sim\rho}\left[\mathbf{1}\{\mu_{\mathbf{v}}\ge 1-\varepsilon/5\}\mathbf{1}\{\text{Tr}\left[(\mathbb{I}-|\Psi\rangle\langle\Psi|)\rho^{A_N}_{\mathbf{w}}\right]> 2\varepsilon/5\}\text{Tr}\left[(\mathbb{I}-|\Psi\rangle\langle\Psi|)\rho^{A_N}_{\mathbf{w}}\}\right]\right]\\ &\le \mathbb{E}_{l,U,(\mathbf{v},\mathbf{w})\sim\rho}\left[\|\rho^{A_N}_{\mathbf{w}}-\rho^{A_N}_{\mathbf{v},\mathbf{w}}\|_1\right] + \frac{2\varepsilon}{5}\cdot\mathbb{E}_{l,U,(\mathbf{v},\mathbf{w})\sim\rho}\left[\mathbf{1}\{\mu_{\mathbf{v}}\ge 1-\varepsilon/5\}\right]\\ &\quad + \mathbb{E}_{l,U,\mathbf{w}\sim\rho}\left[\sum_{\mathbf{v}}\left|\text{Tr}\left[M_{\mathbf{v}}\rho^{A_1\cdots A_k}_{\mathbf{w}}\right]-\text{Tr}\left[M_{\mathbf{v}}\left(\rho^{A_N}_{\mathbf{w}}\right)^{\otimes k}\right]\right|\right]\\ &\quad + \mathbb{E}_{l,U,\mathbf{w}\sim\rho,\mathbf{v}\sim\left(\rho^{A_N}_{\mathbf{w}}\right)^{\otimes k}}\left[\mathbf{1}\{\mu_{\mathbf{v}}\ge 1-\varepsilon/5\}\mathbf{1}\{\text{Tr}\left[(\mathbb{I}-|\Psi\rangle\langle\Psi|)\rho^{A_N}_{\mathbf{w}}\right]> 2\varepsilon/5\}\right]\\ &\le 2\sqrt{\frac{4k^2\log(d)}{N}} + \frac{2\varepsilon}{5} + \sqrt{\frac{4k^2\log(d)}{N}} + \mathbb{E}_{l,U,\mathbf{w}\sim\rho,\mathbf{v}\sim\left(\rho^{A_N}_{\mathbf{w}}\right)^{\otimes k}}\left[\mathbf{1}\{\mu_{\mathbf{v}}-\langle\Psi|\rho^{A_N}_{\mathbf{w}}|\Psi\rangle\ge\varepsilon/5\}\right]\\ &\le \varepsilon\end{aligned}$$

where we set $k = \frac{4e^2 5^2\log(5/\varepsilon)}{\varepsilon^2}$ ([2]), $N = \frac{8^2 k^2\log(d)}{\varepsilon^2}$ and use Equation (10) proven in Lemma 1 of the main text that we recall in the following:

$$\mathbb{E}_{l,\mathbf{r},\mathbf{w},\mathbf{v}}\left[\left\|\rho^{A_N}_{l,\mathbf{r},\mathbf{w},\mathbf{v}}-\rho^{A_N}_{l,\mathbf{r},\mathbf{w}}\right\|_1\right]\le 2\sqrt{\frac{4k^2\log(d)}{N}}.$$

References

[1] Tim van Erven and Peter Harremos. "Rényi Divergence and Kullback-Leibler Divergence". In: *IEEE Transactions on Information Theory* 60.7 (2014), pp. 3797–3820.

[2] Hsin-Yuan Huang, Richard Kueng, and John Preskill. "Predicting many properties of a quantum system from very few measurements". In: *Nature Physics* 16.10 (2020), pp. 1050–1057.
